# Progress Toward Eradication of Dracunculiasis — Worldwide, January 2022–June 2023

**DOI:** 10.15585/mmwr.mm7245a4

**Published:** 2023-11-10

**Authors:** Donald R. Hopkins, Adam J. Weiss, Sarah Yerian, Sarah G.H. Sapp, Vitaliano A. Cama

**Affiliations:** ^1^The Carter Center, Atlanta, Georgia; ^2^Division of Parasitic Diseases and Malaria, National Center for Emerging and Zoonotic Infectious Diseases, CDC.

**Summary**

**What is already known about this topic?**

Human cases of dracunculiasis (Guinea worm infection causing limb pain and disability in some Asian and African countries) have decreased from an estimated 3.5 million in 1986 to 15 in 2021. The emergence of Guinea worm infections in dogs in 2012 has complicated eradication efforts.

**What is added by this report?**

Thirteen human cases and 686 animal infections were reported in 2022. Three human cases and 315 animal infections were reported during January–June 2023. As of August 2023, dracunculiasis remained endemic in five countries (Angola, Chad, Ethiopia, Mali, and South Sudan).

**What are the implications for public health practice?**

With only 13 human cases identified in 2022 and three during January–June 2023, program efforts appear to be closer to reaching the goal of eradication. However, dog infections and impeded access because of civil unrest and insecurity in Mali and South Sudan continue to be the greatest challenges for the program.

## Abstract

The effort to eradicate *Dracunculus medinensis*, the etiologic agent of dracunculiasis, or Guinea worm disease, commenced at CDC in 1980. In 1986, with an estimated 3.5 million cases worldwide in 20 African and Asian countries, the World Health Assembly called for dracunculiasis elimination. The Guinea Worm Eradication Program (GWEP) was established to help countries with endemic dracunculiasis reach this goal. GWEP is led by The Carter Center and supported by partners that include the World Health Organization, UNICEF, and CDC. In 2012, *D. medinensis* infections were unexpectedly confirmed in Chadian dogs, and since then, infections in dogs, cats, and baboons have posed a new challenge for GWEP, as have ongoing civil unrest and insecurity in some areas. By 2022, dracunculiasis was endemic in five countries (Angola, Chad, Ethiopia, Mali, and South Sudan), with only 13 human cases identified, the lowest yearly total ever reported. Animal infections, however, were not declining at the same rate: 686 animal infections were reported in 2022, including 606 (88%) in dogs in Chad. Despite these unanticipated challenges as well as the COVID-19 pandemic, countries appear close to reaching the eradication goal. GWEP will continue working with country programs to address animal infections, civil unrest, and insecurity, that challenge the eradication of Guinea worm.

## Introduction

Dracunculiasis (Guinea worm disease), caused by the parasite *Dracunculus medinensis*, is acquired by drinking water containing small crustacean copepods (water fleas) infected with *D. medinensis* larvae (*1*). Recent evidence suggests that the parasite also might be transmitted by eating inadequately cooked fish or other aquatic animals (*2*). Typically, approximately 1 year after infection, the worm emerges through the skin (usually on the host’s lower limb), causing pain and disability (*1*). No vaccine or medicine is available to prevent or treat dracunculiasis. Eradication relies on case containment* to prevent water contamination and other interventions to prevent infection, including health education, water filtration, treatment of unsafe water with temephos (an organophosphate larvicide), provision of safe drinking water, adequate cooking of aquatic animals intended for consumption, and safe disposal of fish entrails (*1*–*4*). CDC began worldwide eradication efforts in 1980, and in 1984, was designated by the World Health Organization (WHO) as the technical monitor of the Dracunculiasis Eradication Program (*1*). In 1986, with an estimated 3.5 million human cases^†^ occurring annually in 20 African and Asian countries^§^ (*5*), the World Health Assembly called for dracunculiasis elimination. The Guinea Worm Eradication Program (GWEP),^¶^ led by The Carter Center and supported by partners that include WHO, UNICEF, and CDC, began assisting ministries of health in countries with endemic disease. Since 1986, WHO has certified 200 countries, areas, and territories as dracunculiasis-free. Five countries with ongoing endemic dracunculiasis (Angola, Chad, Ethiopia, Mali, and South Sudan), plus Sudan, which has not yet completed its dossier and follow-up visit by WHO, have not been certified by WHO.**

From 2012, a new challenge emerged, posed by animal infections, mostly in domestic dogs, some domestic cats, and in Ethiopia, a few baboons; animal infections have now surpassed human cases. Genetic studies confirmed that Guinea worms from animals and humans are *D. medinensis* (*6*). This report describes progress made during January 2022–June 2023 and updates previous reports (*3*,*7*).

## Methods

Each country’s GWEP provided data on *D. medinensis* infections in humans and animals collected during January 2022–June 2023. Programs receive monthly case reports from supervised volunteers in each village under active surveillance.^††^ Supervisors review the reports of human and animal infections and verify case containment at regional and national levels, where epidemiologic investigation of all human cases and selected animal infections are also analyzed. Specimens requiring laboratory confirmation are sent to CDC’s Laboratory of Parasitic Diseases and Malaria. Villages where endemic transmission has ended (i.e., zero human cases or animal infections reported for ≥12 consecutive months), are kept under active surveillance for 2 additional years. WHO certifies a country as dracunculiasis-free after adequate nationwide surveillance for ≥3 consecutive years with no indigenous human cases or animal infections.^§§^ This activity was reviewed by CDC, deemed not research, and was conducted consistent with applicable federal law and CDC policy.^¶¶^

## Results

### Reported and Laboratory-Confirmed Human and Animal Dracunculiasis Cases Worldwide

During 2022, a total of 13 human dracunculiasis cases were identified worldwide, in Central African Republic, Chad, Ethiopia, and South Sudan; these cases represent a 13% decrease from the 15 cases reported in 2021 (Table 1). During January–June 2023, three human cases were identified (no change from the number reported during the same period in 2022). Angola, Cameroon, Chad, Ethiopia, Mali, and South Sudan reported 686 animal (mostly dog) infections in 2022, a 21% decrease compared with the 862 animal infections reported in 2021. However, the 315 animal infections reported during January–June 2023 represented a 3% increase over the 305 reported during January–June 2022 (Table 2). Epidemiologic investigations identified the probable sources of 85% (11 of 13) of the human cases in 2022 compared with 47% (seven of 15) of cases in 2021.

**TABLE 1:** Reported dracunculiasis human cases and animal infections, surveillance, and status of local interventions in villages with endemic disease, by country — worldwide, 2022

Human cases/Surveillance/Intervention status	Country						
	Chad*	Ethiopia	Mali^†^	South Sudan	Angola	Cameroon	Total
**Reported human cases**							
No. of indigenous	7^§^	1	0	5	0	0	**13**
No. of imported	0	0	0	0	0	0	**0**
% Contained^¶^ (no./total no.)	43 (3/7)	100 (1/1)	NA	60 (3/5)	NA	NA	**54 (7/13)**
% Change in indigenous human cases in villages or localities under surveillance (no. in 2021 vs. 2022)	−13 (8 vs. 7)	0 (1 vs. 1)	−100 (2 vs. 0)	25 (4 vs. 5)	NA (0 vs. 0)	NA (0 vs. 0)	**−13 (15 vs. 13)**
**Reported animal infections**							
No. of indigenous	606	3	41	1	7	28	**682**
No. of imported	0	0	0	0	0	0	**0**
% Contained^¶^ (no./total no.)	70 (421/606)	33 (1/3)	63 (26/41)	100 (1/1)	0 (0/7)	100 (28/28)	**70 (477/682)**
% Change in indigenous animal infections in villages or localities under surveillance (no. in 2021 vs. 2022)	−27 (832 vs. 606)	0 (3 vs. 3)	141 (17 vs. 41)	NA (0 vs. 1)	NA (0 vs. 7)	180 (10 vs. 28)	**−21 (862 vs. 682)**
**Villages under active surveillance, 2022**							
No. (%) of villages reporting monthly	2,434 (98)	411 (100)	2,216 (100)	2,044 (91)	61 (100)	15 (100)	**7,181 (98)**
No. reporting one or more human case	5	1	0	3	0	0	**9**
No. reporting only imported human cases	0	0	0	1	0	0	**1**
No. reporting indigenous human cases	5	1	0	2	0	0	**8**
No. reporting one or more animal infections	265	3	23	1	3	10	**307**
No. reporting only imported animal infections	0	0	0	0	0	0	**0**
No. reporting indigenous animal infections	265	3	23	1	3	10	**307**
**Status of interventions in villages with endemic human dracunculiasis, 2022**							
No. (%) of villages reporting monthly with endemic human dracunculiasis, 2021–2022	14 (86)	2 (100)	1 (100)	7 (100)	0 (NA)	0 (NA)	**24 (92)**
% Filters in all households (no./total no.)	79 (11/14)	100 (2/2)	100 (1/1)	100 (7/7)	NA	NA	**88 (21/24)**
% Using temephos (no./total no.)	79 (11/14)	50 (1/2)	100 (1/1)	100 (7/7)	NA	NA	**83 (20/24)**
% One or more source of safe water (no./total no.)	43 (6/14)	100 (2/2)	100 (1/1)	100 (7/7)	NA	NA	**67 (16/24)**
% Provided health education (no./total no.)	100 (14/14)	100 (2/2)	100 (1/1)	100 (7/7)	NA	NA	**100 (24/24)**
**Status of interventions in villages with endemic animal dracunculiasis, 2022**							
No. of villages with endemic animal dracunculiasis, 2021–2022	509	4	37	1	3	10	**564**
% Reporting monthly (no./total no.)	94 (479/509)	100 (4/4)	100 (37/37)	100 (1/1)	100 (3/3)	100 (10/10)	**95 (534/564)**
% Using temephos (no./total no.)	78 (398/509)	100 (4/4)	100 (37/37)	100 (1/1)	0 (0/3)	100 (10/10)	**80 (450/564)**
% Provided health education (no./total no.)	100 (509/509)	100 (4/4)	100 (37/37)	100 (1/1)	0 (0/3)	100 (10/10)	**99 (561/564)**

**Abbreviations:** GWEP = Guinea Worm Eradication Program; NA = not applicable.

* Participants at the annual Chad GWEP review meeting in November 2014 adopted “1+ case village” as a new description for villages in Chad affected by human cases of Guinea worm disease and dogs infected with Guinea worms and defined it as “a village with one or more indigenous and imported cases of Guinea worm infections in humans, dogs, and/or cats in the current calendar year and/or previous year.”

^†^ Civil unrest and insecurity since a coup in 2012 continued to constrain GWEP operations (i.e., supervision, surveillance, and interventions) in Gao, Kidal, Mopti, Segou, and Timbuktu regions.

^§^ A total of six human cases were reported from Chad in 2022. One human case was reported from the Central African Republic in 2022 in a location along the border with Chad. This case is believed to have been acquired in Chad.

^¶^ Human cases are contained when all of the following criteria are met: 1) infected patients are identified ≤24 hours after worm emergence; 2) patients have not entered any water source since worm emergence; 3) a village volunteer or health care provider properly treats the lesion until all detectable worms are fully removed and educates the patient not to contaminate water sources; 4) the containment process is validated by a GWEP supervisor ≤7 days after worm emergence; and 5) all contaminated and potentially contaminated sources of drinking water are treated with temephos. The criteria for defining a contained case of dracunculiasis in a human also should be applied, as appropriate, to define containment for an animal with a Guinea worm infection.

**TABLE 2:** Number of reported indigenous human and animal dracunculiasis cases, by country — worldwide, January 2021–June 2023

Country	No. of cases (% contained)		% Change Jan–Dec 2021 to Jan–Dec 2022	No. of cases (% contained)		% Change Jan–Jun 2022 to Jan–Jun 2023
	Jan–Dec 2021	Jan–Dec 2022		Jan–Jun 2022	Jan–Jun 2023	
**Human cases**						
Chad*	8 (75)	7 (43)	−13	3 (33)	2 (100)	−33
Ethiopia	1 (100)	1 (100)	0	0 (—)	0 (—)	NA
Mali^†^	2 (50)	0 (—)	–100	0 (—)	0 (—)	NA
South Sudan	4 (25)	5 (60)	25	0 (—)	0 (—)	NA
Angola	0 (—)	0 (—)	NA	0 (—)	0 (—)	NA
Cameroon^§^	0 (—)	0 (—)	NA	0 (—)	1 (100)	NA
**Total**	**15 (60)**	**13 (54)**	**–13**	**3 (33)**	**3 (100)**	**0**
**Animal infections** ^¶^						
Chad	832 (81)	606 (70)	−28	268 (75)	220 (75)	−18
Ethiopia	3 (67)	3 (33)	0	0 (—)	0 (—)	NA
Mali^†^	17 (59)	41 (63)	141	2 (100)	7 (100)	250
South Sudan	0 (—)	1 (100)	NA	0 (—)	0 (—)	NA
Angola	0 (—)	7 (0)	NA	7 (0)	32 (0)	357
Cameroon^§^	10 (100)	28 (100)	180	28 (100)	56 (91)	100
**Total**	**862 (80)**	**686 (70)**	**−21**	**305 (76)**	**315 (71)**	**3**

**Abbreviation:** NA = not applicable.

* Chad’s human case count for January–December 2022 includes a human case detected in an area of the Central African Republic near the border with Chad.

^†^ Civil unrest and insecurity since a coup in 2012 continued to constrain Guinea Worm Eradication Program operations (i.e., supervision, surveillance, and interventions) in Gao, Kidal, Mopti, Segou, and Timbuktu regions.

^§^ One human case and multiple infected animals detected in areas of Cameroon near the border with Chad might have been infected in Chad.

^¶^ Chad: primarily dogs, some cats; Ethiopia: dogs, cats, and baboons; Mali: dogs and cats; Angola: dogs; Cameroon: dogs and cats.

During January–June 2023, CDC received 15 specimens from humans, and only one (7%) was laboratory-confirmed as D. medinensis*** (Table 3), compared with 20 specimens received and three (15%) laboratory-confirmed during January–June 2022. No human cases were reported for an unprecedented period of 6 consecutive months (November 1, 2022–April 30, 2023). During the first 6 months of 2023, CDC received 131 animal specimens, 114 (87%) of which were laboratory-confirmed as *D. medinensis*, compared with 10 (83%) confirmed among 12 specimens received during January–June 2022. 

**TABLE 3:** Characteristics of specimens from humans and animals received at CDC for laboratory confirmation of *Dracunculus medinensis* — January 2022–June 2023

Characteristic	Surveillance period			
	2023	2022		
	Jan–Jun	Jan–Jun	Jul–Dec	Jan–Dec
**Human specimens**				
Country of origin, no. of positive specimens (patients)*				
Central African Republic	—**^†^**	—	1 (1)	1 (1)
Chad	1 (1)	3 (3)	5 (5)	8 (8)
Ethiopia	—	—	1 (1)	1 (1)
South Sudan	—	—	9 (6)	9 (6)
**Total no. (%) of positive specimens**	**1 (7)**	**3 (15)**	**16 (48)**	**19**^§^ **(36)**
**Negative specimens, no. (%) of laboratory identifications**				
Free-living organism^¶^	1 (7)	1 (6)	3 (18)	4 (12)
*Onchocerca* sp.	2 (14)	6 (35)	5 (29)	11 (32)
Other parasitic helminth**	—	—	1 (6)	1(3)
Other parasitic nematode^††^	4 (29)	1 (6)	—	1 (3)
Plant material	—	2 (12)	1 (6)	3 (9)
Sparganum	3 (21)	3 (18)	1 (6)	4 (12)
Tissue (animal origin)	1 (7)	4 (24)	5 (29)	9 (26)
Unknown origin	3 (21)	—	1 (6)	1 (3)
Total no. (%) of negative specimens*	**14 (93)**	**17 (85)**	**17 (52)**	**34 (64)**
**Total no. of human specimens**	**15**	**20**	**33**	**53**
**Animal specimens**				
Positive specimens, country and species of origin, no. of specimens (no. of animals)*				
**Angola**				
Dog	32 (32)	6 (6)	1 (1)	7 (7)
**Cameroon**				
Dog	67 (65)	—	46 (28)	46 (28)
**Chad**				
Cat	—	2 (2)	2 (2)	4 (4)
Dog	8 (7)	—	1 (1)	1 (1)
Other animals (mustelid)	—	—	1 (1)	1 (1)
**Ethiopia**				
Baboon	—	—	2 (2)	2 (2)
Dog	—	—	1 (1)	1 (1)
Other animal (wildcat)	1 (1)	—	—	—
**Mali**				
Cat	—	—	2 (2)	2 (2)
Dog	6 (6)	2 (2)	43 (40)	45 (42)
**South Sudan**				
Dog	—	—	1 (1)	1 (1)
No. (%) of positive specimens*	**114 (87)**	**10 (83)**	**100 (88)**	**110 (88)**
Total no. (%) of negative specimens*	**17 (13)** ^§§^	**2 (17)**	**13 (12)**	**15 (12)** ^¶¶^
**Total no. of animal specimens**	**131**	**12**	**113**	**125**

* Positive specimens were confirmed as *D. medinensis*; negative specimens were ruled out as *D. medinensis.*

^†^ Dashes indicate no specimens received.

^§^ CDC received 19 specimens in 2022: one was from 2021 (Chad), and 18 were from 2022. The specimens from 2022 were from 13 human cases among which 10 had one specimen each, two had two specimens each, and one had three specimens.

^¶^ Free-living organisms primarily included adult Mermithidae and other worms identified as belonging to nonparasitic taxa.

** Other parasitic helminths submitted in association with human cases belonging to the cestode class (flatworms).

^††^ Other parasitic nematodes submitted in association with human cases belonging to the filarioidea or ascarididae families.

^§§^ In 2023, the 17 *D. medinensis*–negative specimens were identified as follows: 10 were other parasitic nematodes from which five were filaroidea, three were *Setaria* sp., and one was *Hastopiculum* sp., and one strongyloidea; one other parasitic helminth was a cestode; two were free-living organisms (mermithids); one was animal tissue, likely from fish; and three samples were of unknown origin.

^¶¶^ The 15 negative specimens from animals from 2022 were identified as follows: eight were other parasitic nematodes from which five were filaroidea, one was *Hastopiculum* sp., one was *Protospirura* sp., and one was nematode; one was another parasitic helminth (a cestode); two were spargana; two were free-living organisms (mermithids); and two were animal tissues (one tendon-like tissue and one case of possibly congealed mucus).

### Country Reports

**Angola.** Angola first discovered Guinea worm cases in 2018 (*8*). Active community-based surveillance began in 54 villages in 2020 and expanded to 61 communities in 2022. Seven infected dogs were detected in 2022, and 32 during January–June 2023 (Table 2), all in the same province as previous infections. Genetic analysis has not linked Angola’s Guinea worms to *D. medinensis* specimens from other countries (Elizabeth Thiele, PhD, Vassar College, personal communication, September 2023). Angola offers a cash reward equivalent to US$450 for reporting an infected human or animal. In 2023, the Angola program began proactively tethering dogs at risk for infection, (i.e., living in or adjacent to villages with endemic disease) and began using temephos in affected areas in June.

**Chad.** Chad reported seven human cases in 2022,^†††^ compared with eight cases in 2021; during January–June 2023, two cases were reported, compared with three during January–June 2022. Chad reported 606 animal infections (521 dogs and 85 cats) in 2022, compared with 832 (767 dogs and 65 cats) in 2021. During January–June 2023, Chad reported 220 infected animals, 18% fewer than the 268 reported during January–June 2022. Transmission of *D. medinensis* in Chad is hypothesized to result from consumption of inadequately cooked aquatic animals including fish or other transport hosts or paratenic hosts^§§§^ (*2*). The Carter Center helped Chad’s GWEP implement village-based surveillance for animal and human infections in 2,434 villages at risk for Guinea worm exposure by December 2022 (Table 1). Active surveillance generated 41,135 rumors (a report of a possible case) about possible Guinea worm infections among humans or animals during January–June 2022; these reports increased 169%, to 110,784 rumors during January–June 2023.

Since 2010, Chad’s Ministry of Health has offered a reward equivalent to US$100 for reporting a confirmed human dracunculiasis case, and since 2015 a US$20 reward equivalent for reporting an animal infection. Evaluations in areas with established active surveillance indicated that 72% and 57% of residents surveyed during 2022 and January–June 2023, respectively, were aware of the rewards.

Chad launched a nationwide campaign in 2013 to increase Guinea worm awareness and implemented educational interventions to prevent transmission through consumption of uncooked fish or fish entrails. Since June 2017, approximately 81% of households assessed monthly in at-risk communities were burying fish entrails to prevent consumption by dogs.

Chad’s GWEP began tethering dogs with dracunculiasis-compatible signs in 2014; in March 2020, it began proactively tethering all dogs during the 4 months of peak dracunculiasis incidence in all villages with five or more dracunculiasis infections during the previous year, increasing to villages with three or more infections in 2021, and to all villages reporting one or more dog infections in the previous or current year in 2022. As a result, 64% and 75% of eligible dogs were tethered in 2022 and January–June 2023, respectively. Water treatment with temephos reached 94% of 280 villages with dog or human infections by December 2022 and 91% of 125 villages by June 2023. In December 2022, 40% of villages reporting Guinea worm infections had at least one source of copepod-free drinking water (e.g., from a borehole well). In June 2023, Chad’s minister of health visited an area with endemic disease to support the Guinea worm eradication program and advocate for eradication.

**Cameroon.** Cameroon reported 28 infected dogs in 2022 and one human infection and 56 confirmed infected dogs during January–June 2023 in villages <3 miles (<5 km) from the Chad-Cameroon border. These Guinea worm infections were likely contracted in Chad because the affected villages include families living on both sides of the border and dog owners take their dogs to Chad regularly. Cameroon expanded active surveillance to all villages of concern and began proactive tethering of dogs in the affected area in January 2022.

**Ethiopia.** Ethiopia reported one human case, two infected baboons, and one infected dog during 2022; and no infected humans or animals during January–June 2023 (Table 2). Ethiopia is the only country that has reported infected baboons. Almost all recent infections have occurred in western Ethiopia, in the Gog district of Gambella Region. With The Carter Center’s assistance, Ethiopia’s public health and wildlife authorities resumed trapping and examining baboons in December 2022 and June 2023. Since 2021, the Ethiopia Dracunculiasis Eradication Program has conducted active surveillance in 198 villages and 223 non-village areas (e.g., farms and fishing and hunting settlements). Surveillance from the examination of baboons found dead or killed by villagers, and from approximately 555 baboons tracked and trapped by researchers (since 2018) detected 20 infected baboons during 2013–2021, two in 2022, and zero in January–June 2023, in an area of about 50 x 25 miles (80 x 40 km) in Gog district and part of adjacent Abobo district.

The reward for reporting human dracunculiasis cases is equivalent to US$360 and for reporting and tethering infected animals is US$40. In 2022, 72% of persons surveyed in active surveillance areas knew of the rewards; in January–June 2023, 97% were aware.

Since April 2018, Ethiopia has supported villager-initiated constant tethering of approximately 1,900 dogs and cats in villages at highest risk, to prevent their exposure in adjacent forests where transmission apparently occurs. The program applies temephos monthly to nearly all water sources known to have been used by humans in the at-risk areas of Gog and adjacent Abobo districts. Beginning in 2022, remote sensing data from Maxar Technologies, a space technology and intelligence company (https://www.maxar.com), has been identifying new water sources that need to be treated. In May 2022, the minister of health visited areas with endemic dracunculiasis to advocate for Guinea worm eradication.

**Mali. **Mali reported no human dracunculiasis cases in January 2022–June 2023, compared with two cases in 2021 (Table 2). In 2022, a total of 41 infected animals were reported, compared with 17 in 2021. During January–June 2023, seven confirmed infections in dogs were reported, all in Segou Region of central Mali, an increase from two reported during January–June 2022. Among the infected animals identified in 2022, 31 were in Segou Region; and 10 in adjacent Mopti Region, which is partly inaccessible because of civil unrest. Animals from Segou Region apparently became infected in Mopti Region. The infections of all seven dogs in January–June 2023 were reportedly contained.

In 2022, a total of 2,216 villages in Mali were under active surveillance (Table 1), with cash rewards equivalent to US$340 for reporting a human case and US$20 for reporting and tethering an infected animal. In areas under active surveillance in 2022, 82% of persons queried knew about the rewards for reporting an infected person or animal; in January–June 2023, 88% knew about the rewards. Proactive tethering of some dogs was introduced late in 2021, expanded during the June–September peak transmission season in 2022, and extended to include puppies in 2023.

**South Sudan.** South Sudan reported five human dracunculiasis cases in 2022, compared with four in 2021 (Table 2). No human cases or infected animals were reported during January 2022–June 2023. Only two infected dogs have ever been reported in South Sudan, the first in September 2015 and the second in August 2022. The high mobility of cattle herders and others in South Sudan poses a challenge to GWEP surveillance and interventions as does sporadic insecurity. By December 2022, a total of 2,044 villages in South Sudan were under active surveillance (Table 1). The reward for reporting a case of dracunculiasis or an infected animal is equivalent to about US$380. A 2022 survey found that 78% of respondents were aware of the reward for reporting an infected person. In April 2023, the minister of health visited an area with endemic dracunculiasis to advocate for Guinea worm eradication.

## Discussion

The 13 human dracunculiasis cases identified in 2022 represented the lowest annual number of human cases ever reported, and no cases were reported for an unprecedented period of 6 consecutive months. Progress toward guinea worm eradication was reviewed at the 2022 and 2023 annual meetings of GWEP program managers, the 2022 meeting of WHO’s International Commission for the Certification of Dracunculiasis Eradication, and an unofficial meeting during the 2023 World Health Assembly. Representatives from seven countries with current or former endemic dracunculiasis, the United Arab Emirates, The Carter Center, and WHO renewed their commitment to completing Guinea worm eradication at a Guinea Worm Summit in Abu Dhabi in March 2022.

### Infections in Dogs

During January 2022–June 2023, Chad reported 82% (835 of 1,017) of the world’s remaining *D. medinensis* human and animal infections, of which 88% (893 of 1,017) were in dogs. Chad reported Guinea worm infections in domestic dogs for the first time in 2012 (*9*), mostly from communities along the Chari River in a pattern that remains peculiar to Chad (i.e., many cases in dogs and few in humans) (*9*,*10*). Stopping transmission among dogs is now the GWEP’s principal challenge.

Guinea worm infections in dogs declined by 24% between 2021 and 2022. The 5% increase in dog infections during January–June 2023 reflects substantial increases in reported dog infections in Angola and Cameroon after expanded active surveillance for dog infections in those countries in 2022. This finding underscores the need for active surveillance, appropriate control measures, and thorough investigations in both countries. In Chad, however, dog infections declined for the third and fourth consecutive years, by 32% from 767 in 2021 to 521 in 2022, and by 17%, from 264 during January–June 2022 to 220 during January–June 2023.

The challenge of animal infections, which occurs in limited geographic areas (except in Chad), is being addressed through innovative interventions and research supported by The Carter Center, WHO, and CDC. After being pioneered by Ethiopia in 2018, proactive tethering of dogs in at-risk villages has proven effective and has been adopted by GWEPs in Chad (2020), Mali (2022), Cameroon (2022), and Angola (2023). Multiple research institutions are working to understand the unusual epidemiology of dracunculiasis in the remaining affected countries and to develop new interventions.

### Infections in Humans and Nonhuman Primates

Finding three confirmed human cases and seven infected dogs in Angola in 2018–2022 and two human cases, 43 infected dogs, and one infected cat in Cameroonian villages that border areas with endemic transmission in Chad during 2019–2022 suggests that the problem in those two countries is limited. Detection of a human case of dracunculiasis in Central African Republic bordering an area with endemic dog infections in Chad also highlights the risks for exportation and the need for ongoing active surveillance and appropriate control measures in neighboring countries. The trend of infections in baboons appears to be declining in Ethiopia because of intensive temephos treatments of water sources in the areas of concern.

Adequate security is critical to achieving eradication, especially in Mali and South Sudan. In 2020, Mali’s GWEP began working with ministry of health, regional, and local leaders in a Peace-Health Initiative to reduce insecurity in one district with endemic dracunculiasis and expanded to four districts in 2022. If adequate security is maintained, South Sudan is poised to achieve zero-case status soon, thanks to strong technical leadership and political support, and no known endemicity in animals.

### Limitations

The findings in this report are subject to at least two limitations. First, the number of Guinea worm infections in humans or animals could have been underestimated, because infections were either undiscovered, not reported, deliberately hidden, or because insecurity impeded program access to some areas. These risks were partially mitigated by wide knowledge of the cash reward for reporting cases and active surveillance by trained local personnel in all areas with known infections. Second, the extent of Guinea worm infection among baboons in Ethiopia, although better understood, is still being determined. 

### Implications for Public Health Practice

With only 13 human cases identified in 2022 and three during January–June 2023, programs appear to be closer to reaching the goal of eradication. However, infections in dogs and impeded access due to civil unrest and insecurity in parts of Mali and South Sudan continue to be the most important challenges for the eradication program.

## Footnotes

* Human cases are contained when all of the following criteria are met: 1) infected patients are identified ≤24 hours after worm emergence; 2) patients have not entered any water source since worm emergence; 3) a village volunteer or health care provider properly treats the lesion until all detectable worms are fully removed and educates the patient not to contaminate water sources; 4) the containment process is validated by a GWEP supervisor ≤7 days after worm emergence; and 5) all contaminated and potentially contaminated sources of drinking water are treated with temephos. The criteria for defining a contained case of dracunculiasis in a human also should be applied, as appropriate, to define containment for an animal with a Guinea worm infection.

† A dracunculiasis case is defined as an infection occurring in a person exhibiting a skin lesion or lesions with emergence of one or more worms that is laboratory-confirmed as *Dracunculus medinensis* at CDC. Because *D. medinensis* has a 10–14-month incubation period, each infected person is counted as having an infection only once during a calendar year.

§ Initially 20 countries, but the former country of Sudan officially separated into two countries (South Sudan and Sudan) on July 9, 2011, and Yemen discovered Guinea worm cases in 1994.

¶
https://www.who.int/activities/eradicating-dracunculiasis

**
https://iris.who.int/bitstream/handle/10665/354570/WER9721-22-eng-fre.pdf?sequence=

†† Villages under active surveillance are those that have endemic dracunculiasis or are at high risk for importation. Active surveillance involves daily searches of households by village volunteers (supported by their supervisors) for persons or animals with signs of dracunculiasis.

§§ An indigenous dracunculiasis human case or animal infection is defined as an infection consisting of a skin lesion or lesions with emergence of one or more Guinea worms in a person or animal who had no history of travel outside their residential locality during the preceding year.

¶¶ 45 C.F.R. part 46.102(l)(2), 21 C.F.R. part 56; 42 U.S.C. Sect. 241(d); 5 U.S.C. Sect. 552a; 44 U.S.C. Sect. 3501 et seq.

*** Specimens are laboratory-identified as *D. medinensis* at CDC by morphologic examination under a microscope or DNA sequencing assays. https://www.cdc.gov/dpdx/dxassistance.html

††† A human case detected in the Central African Republic near the border with Chad is believed to have been imported from Chad and is counted as a case in Chad.

§§§ An intermediate host in which no parasite development occurs but which serves to maintain the viability of larval stages of a parasite.
